# Rhythm-based assessment and training for children with attention deficit hyperactivity disorder (ADHD): a feasibility study protocol

**DOI:** 10.3389/fnhum.2023.1190736

**Published:** 2023-07-31

**Authors:** Hye Jin Shin, Hyun Ju Lee, Dahyun Kang, Johanna Inhyang Kim, Eunju Jeong

**Affiliations:** ^1^Department of Music and Science for Clinical Practice, Graduate School, Hanyang University, Seoul, Republic of Korea; ^2^Department of Pediatrics, Hanyang University Medical Center, Seoul, Republic of Korea; ^3^College of Medicine, Hanyang University, Seoul, Republic of Korea; ^4^Institute of Mental Health, Hanyang University, Seoul, Republic of Korea; ^5^Department of Psychiatry, Hanyang University Medical Center, Seoul, Republic of Korea; ^6^Department of Music Therapy, Graduate School, Ewha Womans University, Seoul, Republic of Korea

**Keywords:** attention deficit hyperactivity disorder, timing ability, rhythm, attention, working memory, electroencephalography

## Abstract

**Background:**

The timing-related deficits in individuals with attention deficit hyperactivity disorder (ADHD) contribute to the symptom-related difficulties and cognitive impairments. Current assessment and training measurement only target specific aspects of the timing ability, highlighting the need for more advanced tools to address timing deficits in ADHD. The aim of this study is to develop and validate a rhythm-based assessment and training (RAT) program, which intends to provide a comprehensive understanding of and enhancement to the time-related abilities of children with ADHD, thereby demonstrating its clinical efficacy.

**Methods:**

We will use randomized crossover trials in this study, with participants being randomly assigned to either start with the RAT and then proceed to cognitive training or start with cognitive training and then proceed to the RAT. Both groups will undergo pre- and post- evaluations. The evaluation will be administered immediately before and after the 4-week training period using diagnostic questionnaires, cognitive evaluation tools, and resting electroencephalography (EEG) measurements. Notably, EEG measurements will be conducted concurrently with the RAT evaluations.

**Discussion:**

This study develops and evaluates the feasibility and effectiveness of a RAT while using EEG measurements to elucidate the underlying therapeutic mechanism of auditory rhythm at varying levels of complexity. The study will investigate the potential of RAT as a supplementary or alternative approach for managing ADHD. The multifaceted data collected will yield valuable insights to customize training agendas based on individual developmental stages and prognoses.

## 1. Introduction

Attention-deficit hyperactivity disorder (ADHD) is a behavioral disorder commonly observed in children and characterized by symptoms such as attention deficit, hyperactivity, and impulsivity (American Psychiatric Association, 2013). ADHD can be categorized into three subtypes: predominantly inattentive (ADHD-I), predominantly hyperactive-impulsive (ADHD-HI), and combined (ADHD-C). ADHD is one of the most common behavioral disorders among children (Felt et al., 2014) and is known to have long-term implications that persist into adolescence and adulthood. Therefore, appropriate intervention is necessary as the symptoms have a significant impact on various aspects of behavioral functions (Millstein et al., 1997; Faraone et al., 2003). ADHD has been associated with several neuropsychological deficits in motor response, cognitive inhibition, sustained attention, and working memory (Barkley, 1997; Stuss and Alexander, 2000; Noreika et al., 2013).

Recent research has highlighted the timing deficits observed in children with ADHD. Individuals with ADHD exhibit deficits in various temporal domain behaviors, such as the ability to perceive and estimate time intervals (Noreika et al., 2013), to prepare responses while considering future consequences (Rubia et al., 2009; Noreika et al., 2013), and cope with the temporal aspect of behavior (Noreika et al., 2013; Hung et al., 2016). In a perceptual timing task, children with ADHD demonstrated lower performance in a discrimination task than children without ADHD (Smith et al., 2002; Radonovich and Mostofsky, 2004; Yang et al., 2007). Reduced accuracy was observed among children with ADHD during the time estimation task in which they were required to calculate stimulus duration and to reproduce the duration by pressing a button (González-Garrido et al., 2008; Khoshnoud et al., 2018). For a motor timing task, adults with ADHD performed with greater variability during a spontaneous tapping task than did adults without ADHD (Kliger Amrani and Zion Golumbic, 2020). Similarly, children with ADHD showed greater tapping variability during a synchronization-continuation task (Zelaznik et al., 2012). Furthermore, in a temporal foresight task, children with ADHD displayed a preference for smaller, more immediate reward over larger long-term reward during delay discounting tasks (Marco et al., 2009).

Timing ability of ADHD have been measured by interval-based and beat-based tasks. Interval-based tasks measure the ability to discriminate, estimate, and reproduce the duration of time intervals, mostly given with two auditory events (Teki et al., 2011). Participants are often asked to discern and identify the relative length of the given two stimuli, indicating which stimulus was shorter or longer (Smith et al., 2002; Toplak and Tannock, 2005; Yang et al., 2007; Lee and Yang, 2019; Anobile et al., 2022) or asked to estimate and subsequently reproduce the length the stimuli following its removal (González-Garrido et al., 2008; Pollak et al., 2009; Khoshnoud et al., 2018; Fontes et al., 2020). Meanwhile, beat-based timing ability measures the ability of the detection of the beat, spontaneous tapping, and synchronization-continuation. Participants are commonly asked to detect a beat in temporal structure (Fujii and Schlaug, 2013; Dalla Bella et al., 2017), to synchronize their tapping with a series of beats presented at isochronous intervals (Fujii and Schlaug, 2013; Dalla Bella et al., 2017; Kliger Amrani and Zion Golumbic, 2020), and to maintain the tapping after the reference beats disappears (Zelaznik et al., 2012; Dalla Bella et al., 2017).

Timing ability of ADHD observed in interval- and beat-based tasks have been identified as a crucial feature of cognitive deficits. In the interval-based timing ability task, Kerns et al. (2001) reported that children with ADHD had larger errors in time reproduction than typically developing children. The errors were positively correlated with the d-prime measure, which is indicative of inattentiveness. Lee and Yang (2019) reported that children with ADHD demonstrated compromised timing ability when presented with stimuli of longer durations, specifically those ranging from 2–10 s. The diminished performance, further, significantly correlated with WM, as evidenced by a positive correlation between WM (i.e., n-back test) and time discrimination task. Notably, after controlling for WM, the performance difference between typically developing children and those with ADHD was not statistically significant. The findings echoed previous findings suggesting a link between WM deficiencies and timing deficits in individuals with ADHD (Barkley, 1997; Pickering, 2006; Kane et al., 2007; Noreika et al., 2013). In tasks involving the beat-based timing abilities, children with ADHD displayed greater variability than their typically developing counterparts when asked to reproduce an isochronous rhythm (Zelaznik et al., 2012; Kliger Amrani and Zion Golumbic, 2020). Additionally, Puyjarinet et al. (2017) found that individuals with ADHD exhibited timing distortions, likely resulting from the difficulties in tracking the beat of rhythm and generating an internal beat. Such findings may provide valuable insights for developing rhythm-based interventions aimed at evaluating and enhancing WM function in individuals with ADHD.

Rhythm, one of the essential components of music, has been vigorously used to evaluate and train the beat-based timing ability. However, its relationship with cognitive abilities of children with ADHD has been sparsely reported. In beat-based assessment, Fujii and Schlaug (2013) developed the Harvard Beat Assessment Test (H-BAT), a battery of four subtests that assess beat perception and production abilities, consisting of the music tapping test (MTT), the beat saliency test (BST), the beat interval test (BIT), and the beat finding and interval test (BFIT). The MMT measures the ability to tap along with the beat (i.e., the degree of tapping synchronization), the BST measures the ability to discern the degree of beat saliency and tap along with the beat (i.e., the threshold of beat processing), the BIT measures to discern and adopt the tapping along with the changes in tempo, and the BFIT measures the ability to discern and adopt the tapping the rhythmic sequences along with the changes in tempo. Dalla Bella et al. (2017) developed the Battery for the Assessment of Auditory Sensorimotor and Timing Abilities (BAASTA) that include time perception and production tasks. The rhythm perception task consists of two-tone duration discrimination, regularity detection in rhythm sequence, and beat alignment, while the production task consists of free tapping, paced tapping with metronome, and synchronization and continuation after the removal of metronome, and adoptive tapping to changes.

In beat-based training, Bégel et al. (2018) developed a rhythm skill training, titled Rhythm Workers, that consists of (1) discrimination of whether the given beats were aligned with those embedded in music (perception), and (2) reproduction along to the beat embedded in various type of music (tapping). After 2 weeks training program, with sessions occurring five times per week, each lasting 30 minutes, participants in both training groups showed significantly better performance than the control groups as measured by the beat alignment and paced tapping of the BAAST. In another study, Thomson et al. (2013) compared the effect of phonemic and rhythm-based intervention on phonological skills and literacy ability for children with dyslexia. After 6 weeks training, with sessions occurring once a week lasting 30 min, participants in both intervention groups showed significant improvement in phonological processing ability, indicating that rhythm-based training may have the potential for developing the phonological skills necessary for efficient literacy acquisition.

Research using beat-based training has uncovered a potential relationship between changes in rhythmic performance and cognitive ability. Ireland et al. (2018) investigated the relationship between rhythm tapping and cognitive abilities (i.e., attention, WM). School-aged children were trained with (1) the children’s Rhythm Synchronization Task (c-RST) in which children were instructed to tap in synchrony to different types of metrical rhythm (strongly metric, medium metric, weakly metric), and (2) the children’s Melody Discrimination Task (c-MDT) in which children were asked to indicate the similarity in keyness of two melodies. The results showed c-RST was significantly correlated with Digit Span Test (DST) depending on the complexity of melody: simple melodies were strongly associated with DST, whereas transposed melodies were related to letter-number sequencing, which is an indicator of working memory and executive control. In a subsequent study, Bégel et al. (2022a) reported that children with dyslexia had inferior predictive timing performance when compared to their non-dyslexic counterparts, as assessed by BAASTA. Participants exhibited a noticeable decline in motor and cognitive functions, including attention, working memory, and cognitive flexibility, which was significantly correlated with their predictive performance. Further regression analysis indicated that the deficits in predictive timing could partially account for the observed phonological deficit, suggesting the potential for BAASTA to serve as a diagnostic tool for developmental dyslexia. Additionally, Bégel et al. (2022b) found improvement of cognitive flexibility as well as rhythm perception and reproduction in children with developmental cerebellar anomalies as a result of dance training, suggesting a possible relationship between beat processing ability and cognitive flexibility.

Collectively from the previous research findings, some concerns arise when evaluating the current state of research. First, previous studies have not fully grasped the timing ability in ADHD due to the restricted use of stimulus lengths and patterns (Grahn and Schuit, 2012; Fujii and Schlaug, 2013; Dalla Bella et al., 2017). Previous tasks have employed either short or relatively longer independently (Boltz, 2005; Matthews and Meck, 2014; van Rijn, 2018; Riemer et al., 2021), an isochronous beat pattern (Fujii and Schlaug, 2013; Dalla Bella et al., 2017) and rhythmic sequences with limited variation (Jerde et al., 2011; Schaal et al., 2015). Some studies have attempted to introduce complex rhythm metrics, yet their methodology did not involve random generation to generate various levels of complexity (Bégel et al., 2018; Ireland et al., 2018). The second concern pertains to the tasks employed, which largely focused on beat detection and synchronization, without adequately addressing active maintenance and recall. While the synchronization to an incoming rhythm involves sensory-motor integration relying on memory system (Grahn and Rowe, 2009), it seems distinct from active maintenance for recall. Considering the broad spectrum of information complexity expected to be processed, it is needed to employ a comprehensive representation of rhythmic sequences and to utilize tasks that represent the ability to hold, manipulate, and recall. The consideration is particularly significant given the deep connections between timing-related information processing and reading (Friedman et al., 2017; Friedman et al., 2017), language skills (Cohen et al., 2000; Kim and Kaiser, 2000), and memory (Kofler et al., 2011, 2018), which are known to be deficient in children with ADHD.

In the present study, we will develop a rhythm-based assessment and training (RAT) that was designed to reflect the complexity of real-world rhythm processing. Four types of rhythmic sequences were generated based on a series of investigations that employed different levels of complexity in rhythmic stimuli (Essens and Povel, 1985; Grahn and Brett, 2007; Chen et al., 2008; Bengtsson et al., 2009). The types of rhythmic sequences are isochronous (ISO), syncopated (SYN), metric (M), and non-metric (NM) and thus, can serve as an assessment and training tool with an infinite array of test items tailored to individual abilities. Also, we will comprehensively investigate the timing ability in ADHD as measured by music indicators of the RAT and explore its relationship with ADHD symptoms and cognitive abilities as measured by diagnostic questionnaires and cognitive evaluation measurements. Electroencephalography (EEG) will be recorded to scrutinize each phase of information processing, including perception, maintenance and manipulation, and reproduction. Our hypotheses include: (1) RAT performance would significantly differ by the level of rhythmic complexity, (2) music indicators would be significantly correlated with ADHD diagnostic questionnaires and cognitive evaluation measurements, (3) band frequency activities of EEG would significantly differ by the phase of information processing, and (4) behavioral outcome measures would significantly differ by intervention types (i.e., rhythm-based training, cognitive training).

## 2. Methods

### 2.1. Study design

**FIGURE 1:**
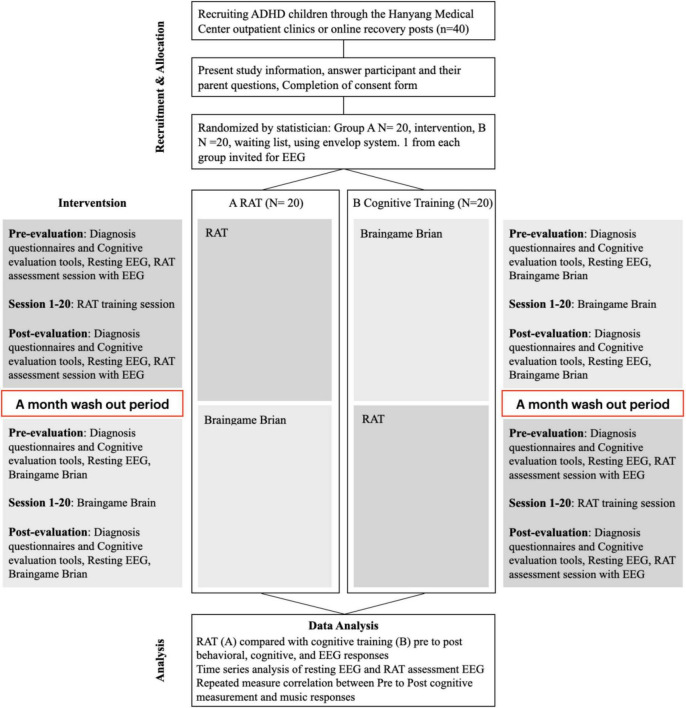
Study scheme showing the crossover design with repeated measures. RAT, rhythm-based assessment and training; EEG, electroencephalography.

Randomized crossover trials, clinical experiments in which participants are assigned randomly to a sequence of treatments and each participant serves as one’s own control in estimating treatment effect (Senn, 2002; Piantadosi, 2005), will be used in the present study. Participants will be assigned randomly to either rhythm training followed by cognitive training or cognitive training followed by rhythm training (Figure 1). For both groups, pre- and post-training evaluations will be administered immediately before training and after the 4th week of training. Participants will have a 4-week washout phase between trainings to minimize a possible carryover effect (Senn, 2002; Piantadosi, 2005). Pre- and post-training evaluations will include diagnosis questionnaires, cognitive evaluation tools, and resting EEG measurement.

### 2.2. Procedure

Before the start of the experiment, the participants and their parents will be provided both oral and written information regarding the purpose of the study and will provide written informed consent. Each participant will be randomly assigned to one of two groups and will undergo pre- and post-training evaluations immediately before and after the 4-week training period. In the evaluation session, the participants will proceed in the order of diagnosis questionnaires, cognitive evaluation tools, and EEG recordings. In the training session, participants will undergo a 4-week RAT or a cognitive training program. The program will be conducted five times a week, with each session lasting 30 min per day. After a 1-month washout period, the training session schedule will be switched between the two groups.

### 2.3. Recruitment

Participants who have met the eligibility criteria will be enrolled in the study after they provide informed consent. Participants and their parents will receive oral and written information detailing the purpose of the study. All experimental protocols will be approved by the Institutional Review Board of Hanyang University Medical Center. Approximately 40 children with ADHD will be recruited through Hanyang Medical Center outpatient clinics or online recruitment posts. The criteria to be included in the examination are as follows: (1) 8 ≤ age ≤ 15; (2) satisfied the criteria of ADHD based on *Diagnostic and Statistical Manual of Mental Disorders 4th edition* (DSM- IV) and diagnosed with ADHD based on the *Korean version of Kiddie-Schedule for Affective Disorders and Schizophrenia-Present and Lifetime* (K-SADS-PL) in a semi-structured clinical interview conducted by a single board-certified psychiatrist; (3) intelligence quotient (IQ) score above 70; (4) have not received medication for ADHD treatment in the past or have received medication in the past but for less than 1 year and not within 4 weeks of the study. The exclusion criteria are as follows: participants with (a) congenital hereditary problems; (b) acquired brain injury such as cerebral palsy; (c) spasmodic disorder, other neurological disorders, or untreated dysesthesia (sensory disturbance); (d) diagnostic history or current state of schizophrenia and childhood psychosis; (e) intellectual disability; and (f) comorbid condition with obsessive-compulsive disorder, depressive disorder, or bipolar disorder. Comorbid conditions, such as anxiety or enuresis, will be allowed except for the symptoms in exclusion criteria. In addition, participants could apply the medication targeting other types of coexist pathologies, not with the symptoms which meet the exclusion criteria.

### 2.4. Intervention

#### 2.4.1. Rhythm-based assessment and training

**FIGURE 2:**
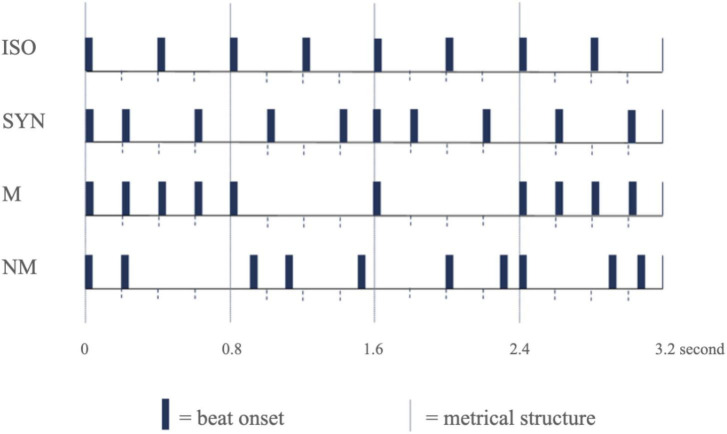
Example of rhythmic sequence subtypes. ISO, isochronous; SYN, syncopated; M, metric; NM, non-metric.

The rhythm-based assessment training (RAT) is a tool using rhythmic sequences for measuring the timing ability in children with ADHD. The four types of auditory rhythmic sequence, named isochronous (ISO), syncopated (SYN), metric (M), and non-metric (NM) (Povel and Essens, 1985), were selected from previous studies examining the behavioral and neural responses to rhythm perception and reproduction (Essens and Povel, 1985; Grahn and Brett, 2007; Chen et al., 2008; Bengtsson et al., 2009; Nozaradan et al., 2011, 2012; Figure 2). The auditory stimuli tones that comprise the rhythmic sequences are of 990 Hz with a duration of 55 ms pure tone (10 ms of each rise and fall time is included) was created using Audacity software (Audacity Team, Pittsburgh, PA, USA, Version 2.3.3). ISO refers to a series of beats that recurs regularly over time (Bengtsson et al., 2009). SYN refers to a rhythmic sequence with displacement of a regular accent associated with a given metrical structure (Longuet-Higgins and Lee, 1984; Sioros et al., 2012; Makris et al., 2022). M refers to a rhythmic sequence with integer ratios with a regular downbeat, and NM refers to a rhythmic sequence with non-integer ratios with irregular downbeats (Grahn and Brett, 2007; Chen et al., 2008; Bengtsson et al., 2009). The four types of rhythmic sequences were randomly generated by using E-Prime software (Psychology Software Tools, Pittsburgh, PA, Version 3.0).

The RAT will include assessment and training sessions. The assessment session will include two types of tasks: (1) a maintenance task in which participants will be asked to listen to the four types of rhythm sequences and then to mentally rehearse the sequences, and (2) a reproduction task in which participants will be asked to listen to the four types of rhythm sequences and then to tap the sequence (Please note that EEG will be concurrently measured during the assessment session. Participants will be instructed to press a button once they have completed the maintenance task, which will then initiate the next trial). A total of 40 trials of rhythmic sequences, 10 trials for each of the four rhythmic sequences, will be randomly presented in each task. In the assessment session, a trial will consist of (1) baseline, (2) rhythm presentation, (3) baseline, (4) cue, and (5) task performance (maintenance, reproduction). In the rhythm presentation, each rhythmic sequence will be presented three times with a 2.5-s interval to provide participants with more opportunities to encode temporal information accurately (Grahn and Brett, 2007).

The training session will consist of practice and main session. In the practice session, the participants will undergo eight practice trials, two trials for each of the four rhythmic sequences to be familiarized with the stimuli and task. In the main training session, participants will be asked to listen to, mentally rehearse the sequences, and tap the rhythmic sequences. A total of 60 trials of rhythmic sequences will be presented; however, the number of rhythmic sequences given to each of the four types of rhythmic sequence will vary upon the individual baseline ability. In the training session, a trial will consist of (1) rhythm presentation, (2) cue, (3) rhythm maintenance, (4) cue, and (5) rhythm reproduction. Table 1 presents overall structure of the RAT, tasks trials assigned across rhythmic sequence and session types (Please note that the number of trials assigned to each of the four rhythmic sequences in training provided is an example; the actual number of training trials will be determined based on the results of the assessment). The rhythmic sequences used in the assessment and training sessions will not overlap with each other. The RAT performance will be evaluated using the numbers of taps, the timing of taps, and the intervals between taps (i.e., tap onset to onset interval, TOA) as compared with those of the original rhythmic sequences. Presentation of RAT and recording of participants’ behavioral responses will be performed using E-prime software (Psychology Software Tools, Pittsburgh, PA, Version 3.0). At the end of each session, participants will be given a one-page performance evaluation report. A total time to completion will be approximately 30 min for both assessment and training.

**TABLE 1:** Overview of RAT structure and task-trial assignment.

Session type	Task type	Number of trials in rhythmic sequence type			
		ISO	*M*	SYN	NM
Pre- Assessment	Maintenance/Reproduction	10/10	10/10	10/10	10/10
Training week 1	Maintenance-Reproduction	24	24	6	6
Training week 2	Maintenance-Reproduction	18	24	12	6
Training week 3	Maintenance-Reproduction	12	18	18	12
Training week 4	Maintenance-Reproduction	6	12	24	18
Post- Assessment	Maintenance/Reproduction	10/10	10/10	10/10	10/10

#### 2.4.2. Cognitive training

In this study, we will utilize Braingame Brian (Prins et al., 2013), a game-based cognitive training consisting of three types of tasks that aim at enhancing the abilities of WM, inhibition, and cognitive flexibility. The WM training task (Dovis et al., 2008) is designed to training manipulate, update, and retain a certain amount of information. Children are presented with the series of visual sequences (i.e., rectangles), and asked to reproduce the sequence by clicking a computer mouse before a subsequent sequence appears. The sequence length (i.e., the number of rectangles) is adjusted to vary based on individual performance.

The inhibition training task, also based on (Dovis et al., 2008), focuses on training inhibitory control of prepotent responses. During the go trials, children are presented with green lights either on the left or right side, and instructed to press the corresponding button within a time window of 700–1,000 ms. During the no-go trials, children are presented with red lights on either side, and asked to withhold pressing the button. The presentation time for red lights is adapted based on individual performance. The cognitive-flexibility training task, adapted from Karbach and Kray (2009), targets executive functioning. Children are tasked with sorting objects based on a given cue (i.e., shape, color) within a time of 1,300 ms. They are also asked to adapt strategies as the cue changes. The response time is adapted based on individual performance. For example, if a child struggles to initiate sorting, the time window allows for a longer duration.

### 2.5. Outcome measures

The diagnosis questionnaires and cognitive evaluation tools are used to analyze the associations between the RAT results and the clinical problems seen in children with ADHD. Through these diagnosis questionnaires and cognitive evaluation tools, information on externalization problems, impulsivity and hyperactivity, inattention, and WM in children with ADHD will be collected.

#### 2.5.1. Diagnosis questionnaires

##### 2.5.1.1. Korean version of the child behavior checklist

The child behavior checklist (CBCL) is a standardized child behavior assessment tool that allows parents to evaluate various aspects of child behavior based on their observations (Achenbach, 1991). In this study, the Korean version of CBCL (K-CBCL) will be used (Oh et al., 1997). The K-CBCL is a parent-report questionnaire with 121 items providing data on various emotional and behavioral problems found in children (Park et al., 2014). The K-CBCL is composed of a social functioning scale and problematic behavior scale. The social functioning scale includes socialization, academic functioning, and total social competence. The problematic behavior scale includes subscales of Withdrawn, Somatic complaints, Anxious/Depressed syndrome, Social problems, Thought problems, Attention problems, Delinquent behavior, Aggressive behavior, Internalizing symptoms, Externalizing symptoms, Total score, Sexual problems, and Emotional Lability. For each item, parents measure child’s behaviors on a three-point scale as follows; they check “0” if their child has never shown the specific behaviors; check “1” if their child shows the behavior somewhat or sometimes; and check “2” if the specific behavior is observed very often. The raw scores for each subscale are calculated by summing the scores obtained from each item, and they are converted to a T score.

##### 2.5.1.2. Disruptive behavior disorder scale

The disruptive behavior disorder scale (DBDS) is a psychiatric assessment scale based on the DSM-IV diagnostic criteria to rate attention deficit/hyperactivity disorder (ADHD), oppositional defiant disorder (ODD), and conduct disorder (CD) (Silva et al., 2005). The Korean version of DBDS will be used for assessment of the disruptive behavioral symptoms of the participants (Kim, 2002). The scale consists of 41 items, including 18 items asking about ADHD symptoms, eight questions about ODD symptoms, and 15 questions about CD symptoms. Each item is calculated on a four-point Likert scale of “0” for “not at all,” “1” for “just a little or sometimes,” “2” for “pretty much or quite often,” and “3” for “very much.” Clinical symptoms were considered if they met the criteria of DSM-IV, at least six of the nine inattentive and/or hyperactive/impulsive symptoms, four of the eight oppositional defiant behaviors, and three of the 15 conduct behaviors (Park et al., 2013).

##### 2.5.1.3. Inattention/overactivity with aggression conners rating scale

The inattention/overactivity with aggression (IOWA) conners rating scale is one of the widely used measures to evaluate the therapeutic effectiveness of ADHD children and has the advantage of being reliable in evaluating various externalizing behaviors such as aggression and rebellious behaviors in ADHD children as well as hyperactivity (Shin et al., 2005; Waschbusch and Willoughby, 2008). The IOWA Conners Rating Scale consists of 10 items about inattentive, impulsive, and overactive (IO) symptoms and oppositional defiant (OD) symptoms. The first five items compose the IO subscale, and the other five items compose the OD subscale. Within the IO subscale, three items measure hyperactivity and impulsivity, and the remaining two measure inattentiveness (Waschbusch and Willoughby, 2008). Each item is calculated using a four-point Likert Scale with “0” for “not at all,” “1” for “just a little,” “2” for “pretty much,” and “3” for “very much.” Total sum of the scores for each questionnaire is calculated and interpreted as the severity of clinical symptoms. Korean version of IOWA Conners rating scale was applied in this study (Shin et al., 2005).

##### 2.5.1.4. Korean version of ADHD rating scale-IV

Korean version of ADHD rating scale-IV (K-ARS-IV) will be used to identify the severity of ADHD symptoms for each participant. The original scale was developed by DuPaul (1991) based on the diagnostic criteria of ADHD described in DSM-IV, and it has been probed that Korean version has high reliability and validity to discriminate and identify the children with ADHD (Kim et al., 2003). The scale is consisted of 18 questionnaires. Parents of participants will rate each questionnaire from 0 to 3, based on the frequency of the children’s problematic behaviors over the past 6 months. The total sum indicates the severity of ADHD symptoms, and the cut-off score for ADHD criteria is 19 for total sum score.

#### 2.5.2. Cognitive evaluation tools

##### 2.5.2.1. Korean version of the wechsler intelligence scale for children: 4th ed.

The Korean version of the wechsler intelligence scale for children: 4th ed. (K-WISC-IV) was used to assess the intellectual and cognitive abilities of the children. The Wechsler intelligence scale is the most widely used tool to assess the intellectual functioning of children and adolescents, and the Korean version was standardized on a sample of 2,448 Korean children, aged from 6–16 years (Kwak et al., 2011). The K-WISC-IV consists of 10 core subtests and five supplemental subtests. The full-scale IQ score (FSIQ) is calculated by the core subtests; using the composite of two or three core subtests, four main indices are calculated: the verbal comprehension index (VCI), perceptual reasoning index (PRI), working memory index (WMI), and processing speed index (PSI). In this study, IQ scores were used for screening the participants, and the digit span (DS) and the letter-number sequencing (LN), which is a subtest of the working memory index. The DS consists of digit span forward (DSF) and digit span backward (DSB) tests. Participants will recall a spoken sequence of numbers in the same order as they were presented (DSF) and in inverse order (DSB). The LN task asked participants to listen to a sequence of numbers and letters arranged in a random order and to recall the numbers in ascending order and then the letters in alphabetical order.

##### 2.5.2.2. Stroop color and word test children’s version

Stroop color and word test (SCWT) is a neuropsychological assessment tool for evaluating the efficiency of the prefrontal lobe, especially attentional and executive functioning (Golden and Golden, 2002). This tool is frequently used for evaluating cognitive inhibition in children (Archibald and Kerns, 1999). The SCWT is composed of three subtest conditions of word, color, and color-word. In the first subtest, word condition, participants should read and speak the colored words printed in black ink. In the color subtest, participants were asked to read the bar of X’s, colored in red, blue, or green ink. Last, in the color-word subtest, colored words are printed a color incongruent with the printed name. Participants should ignore the letter and read the printed ink sequentially as in other subtests. For each subtest, participants should read the stimulus as quickly and accurately as possible within 45 s, and the raw scores will be scored according to the number of correct answers. Interference score is calculated as the difference between the color score and the color-word score. In the current study, the Korean version of SCWT was conducted through the E-Prime software (Psychology Software Tools, Pittsburgh, PA, Version 3.0).

##### 2.5.2.3. Children’s color trails test

The children’s color trails test (CCTT) is a neuropsychological test that can evaluate frontal lobe function of child psychiatric disorders, including attention and executive function (Koo and Shin, 2008). Based on the adult version of the trail making test (TMT), the child’s version minimizes the disadvantages caused by age by avoiding dependence on letters and minimizes the influence of culture and language using colors and numbers instead of letters (Williams et al., 1995). The Korean version of standardized CCTT (Koo and Shin, 2008) was conducted during the study. The test consisted of two subtests: CCTT-1, which measures sequential processing power and psychomotor speed, and CCTT-2, which examines continuous divisional attention and cognitive flexibility (Reum et al., 2021). Under the researcher’s instruction, participants performed each subtest in consecutive order, performing a practice task prior to the actual task. In the first subtest, CCTT-1, participants should connect the numbers 1–15 as quickly and correctly as possible, without lifting the pencil from the paper. For the next subtest, CCTT-2, identical numbers are embedded in separate pink and yellow circles. Participants should connect the numbers sequentially from 1 to 15, alternating between colored circles. The raw score is calculated as the time for completing each task, and the number of errors committed was counted for ascending numbers or altered colors.

##### 2.5.2.4. Advanced test of attention

In this study, we will use the advanced test of attention (ATA), a computerized continuous performance test (Shin et al., 2000) to assess participants’ inattention and inhibitory control. The test consists of visual and auditory tasks. A total of three non-verbal stimuli will be presented randomly during each visual and auditory task, all of which were non-target except for one stimulus. Participants should press the button for the target stimuli as fast as possible, and not respond to the non-target stimuli. For the visual task, an image with a black triangle inside the colored square will be the target stimulus, and for the auditory task, three consecutive beep sounds will be a target stimulus. Each task will be implemented after the practice trial with the researcher. The test will be measuring four main variables: (1) omission error which measures the symptoms of inattention, (2) commission error which measures impulsivity and disinhibition, (3) mean time for correct responses which measure the processing speed, and (4) standard deviation of response time which measures the consistency of attention. Based on normative distribution according to age and gender, all scores will be translated into T scores with average of 50 and standard deviation of 15. In addition, two variables will be also collected: (1) d’ variable to measure whether participants discriminate target stimuli with non-target stimuli well, and (2) Beta (β) to measure the participants’ response bias whether there are more omissions or commissions in the event of error responses. The visual and auditory tasks each lasting 15 min.

##### 2.5.2.5. Comprehensive attention test

The comprehensive attention test (CAT), a Korean version of a computerized continuous performance task will be administered (Yoo et al., 2009). For each selective task, participants will be instructed to make the response to target stimuli and inhibit the response to non-target stimuli. Participants’ performance will be assessed by four main variables: (1) omission errors to measure inattention, (2) commission errors to measure impulsivity, (3) response time to measure the processing speed, and (4) response time standard deviation to measure the variability of the response time. All raw scores will be standardized into attention quotients (AQs), adjusted for age and gender by comparison with a normal population, and the average AQ is 100 with standard deviation of 15. In addition, two variables will be also collected: (1) d’ variable to measure whether participants discriminate target stimuli with non-target stimuli well, and (2) Beta (β) to measure the participants’ response bias whether there are more omissions or commissions in the event of error responses. The visual and auditory selective tasks each lasting 11 min.

### 2.6. EEG

The EEG will be recorded during resting and the RAT assessment sessions. The Nihon Kohden Neurofax EEG device (EEG-1250, Nihon-Kohden, Tokyo, Japan) using 19ch Ag/AgCl electrodes is intended for use in this study. The researchers will observe and record participants’ behavior during the EEG for artifact control, and the records will include the participants’ body movements and eye blinks. Data pre-processing and analysis will use the EEGLAB toolbox for MATLAB.^1^ Artifacts will be excluded using an independent component (ICA) analysis after visual inspection. The EEG data are analyzed with MATLAB^®^ and Simulink^®^ software. The pre-processed EEG data will be used to calculate the power spectral density (PSD) and phase locking value (PLV) for raw and z-scored values.

Power spectral density analysis is performed for each frequency band, and absolute power is used to calculate topography, especially alpha and beta. For topography, especially the upper alpha range (8–40 Hz), we expect that activation in the frontal and posterior regions will differ depending on the type of rhythmic sequence and the type of task. Topography will visually explore the increase or decrease in spectral power. We expect the spectral power to increase, especially in the posterior region, as the rhythmic sequence and task become more complex. Studies have reported an increase in posterior alpha activity during music perception and imagination and have revealed that alpha activity increases with the complexity of music (Günther et al., 1991; Ruiz et al., 2009) and the demand for tasks (Cooper et al., 2003). Beta, which is strongly related to motor activity (Salmelin et al., 1995; Grahn, 2012), and gamma, which is related to attention and memory (Jensen et al., 2007; Grahn, 2012), also selected as our frequency bands of interest.

Phase locking value analysis has been widely used as a robust method for quantifying functional connectivity between brain regions (Lachaux et al., 1999; Mormann et al., 2000; Wang et al., 2020). Compared to visual WM, which has been studied extensively, auditory WM has not been studied as well, and the results are unclear. Several studies have shown theta synchronization as a visual and auditory WM marker (Knyazev, 2007; Düzel et al., 2010; Albouy et al., 2017). In typically developing adults, connectivity in the theta frequency band increases between frontal and parietal regions (Albouy et al., 2017) and between prefrontal and mediotemporal regions (Fell et al., 2008; Fell and Axmacher, 2011) during auditory WM tasks. During the RAT, specifically the maintenance and reproduction tasks, we expect increased connectivity between frontal and parietal and between frontal and medio-temporal regions in the theta band in children with ADHD.

### 2.7. Statistical analysis

All statistical analyses will be conducted using SPSS statistical software (version 24.0, SPSS, Inc., Chicago, IL, USA). The study will undertake a comparative analysis of two intervention sequences and investigate the existence of carry-over effects. This will involve conducting *t*-tests on the change of each sequence for all crossover endpoints (Wellek and Blettner, 2012; Jones and Kenward, 2014). For variables that do not show significant carry-over effects, we will compare the changes during specific interventions in both sequences. For variables that show significant carry-over effects, we will compare the changes in specific interventions during the first period. To confirm the normal distribution of the data, we will analyze the skewness and kurtosis and perform the Shapiro-Wilk and Kolmogorov–Smirnov tests. Firstly, the differences between rhythmic sequences will be compared using repeated measures ANOVA (RMANOVA) and the Bonferroni correction for pairwise comparisons. If the data do not satisfy a normal distribution, Friedman’s test one-way analysis of variance (ANOVA) will be performed. Secondly, Pearson’s correlation test will be used to calculate the correlations between RAT performance and behavioral outcome measures. Thirdly, differences of EEG band activities between the types of rhythmic sequences and the tasks will be compared using RMANOVA with Bonferroni correction. Lastly, comparisons between interventions will be performed using a two-tailed *t*-test or Mann–Whitney U test depending on normality.

## 3. Discussion

The current study aims to develop RAT as a tool for assessing and training the time-related abilities of children with ADHD using a wide range of auditory stimuli organized in various types of rhythmic sequences. Additionally, the study will validate rhythm-based attention training for children with ADHD and its correlations with the behavioral diagnostic questionnaire and scores of cognitive evaluation tools for ADHD. In addition, they study will examine the efficacy of the training in comparison with general cognitive training. The use of EEG will aid in uncovering the nature of auditory rhythm processing in ADHD and in revealing the therapeutic mechanism of auditory rhythm at various levels of complexity. The present findings will contribute to the potential of rhythm-based training as a supplementary or alternative method for behavioral symptoms and cognitive functions of ADHD. The multifaceted data collected will provide valuable insights into the development of a computerized rhythm-based cognitive training program for ADHD and will enable tailored training agendas based on individual developmental stages and prognoses.

The RAT might capture a more comprehensive picture of an individual’s timing ability and potentially lead to more personalized and effective interventions in various clinical and educational contexts. Firstly, the present study could potentially establish the RAT as a new music-based intervention for managing timing-related problems in ADHD. The specific study protocol enables music therapists to measure and quantify musical responses and clearly indicates how changes in musical responses are related to and can lead to symptom-related changes and cognitive enhancement. The behavioral data collected in this study could enable more tailored music-based interventions and, thus, improve the effectiveness of interventions and patient outcomes. Also, by using EEG measurements, the study may provide novel insights into the underlying neural mechanisms of how auditory rhythm can impact cognitive functioning for children with ADHD. This would expand the therapeutic arsenal available to clinicians, offering a non-pharmaceutical intervention that could be used either independently or in conjunction with other treatments. Given the mutual enhancement of WM and cognitive flexibility through rhythm and verbal tasks, an inclusive strategy could improve a deeper understanding between rhythm-related timing ability and cognitive functions.

## Ethics statement

The studies involving human participants were reviewed and approved by the Institutional Review Board of Hanyang University Medical Center (Approval No. 2020-02-025). Written informed consent to participate in this study was provided by the participants’ legal guardian/next of kin.

## Author contributions

HS and EJ developed the protocol, conducted the literature review, and drafted the manuscript. EJ, JK, HL, and DK advised on the experimental design, ethics, and clinical training. JK facilitated the hosting of the study and advised on recruitment sites and procedures. All authors provided critical feedback and edited the manuscript.

## Footnotes

1
http://sccn.ucsd.edu/eeglab
